# Modulation of Differentiation of Embryonic Stem Cells by Polypyrrole: The Impact on Neurogenesis

**DOI:** 10.3390/ijms22020501

**Published:** 2021-01-06

**Authors:** Kateřina Skopalová, Katarzyna Anna Radaszkiewicz, Věra Kašpárková, Jaroslav Stejskal, Patrycja Bober, Ita Junkar, Miran Mozetič, Zdenka Capáková, Marián Lehocký, Martina Kašparová, Jiří Pacherník, Petr Humpolíček

**Affiliations:** 1 Centre of Polymer Systems, Tomas Bata University in Zlín, 760 01 Zlín, Czech Republic; skopalova@utb.cz (K.S.); vkasparkova@utb.cz (V.K.); capakova@utb.cz (Z.C.); lehocky@utb.cz (M.L.); m3_kasparova@utb.cz (M.K.); 2 Department of Experimental Biology, Faculty of Science, Masaryk University, 625 00 Brno, Czech Republic; 432029@mail.muni.cz; 3 Faculty of Technology, Tomas Bata University in Zlín, 760 01 Zlín, Czech Republic; 4 Institute of Macromolecular Chemistry, Academy of Sciences of the Czech Republic, 162 06 Prague 6, Czech Republic; stejskal@imc.cas.cz (J.S.); bober@imc.cas.cz (P.B.); 5 Department of Surface Engineering, Jožef Stefan Institute, Jamova cesta 39, SI-1000 Ljubljana, Slovenia; ita.junkar@ijs.si (I.J.); miran.mozetic@ijs.si (M.M.)

**Keywords:** conducting polymer, polypyrrole, biocompatibility, neurogenesis, stem cells

## Abstract

The active role of biomaterials in the regeneration of tissues and their ability to modulate the behavior of stem cells in terms of their differentiation is highly advantageous. Here, polypyrrole, as a representantive of electro-conducting materials, is found to modulate the behavior of embryonic stem cells. Concretely, the aqueous extracts of polypyrrole induce neurogenesis within embryonic bodies formed from embryonic stem cells. This finding ledto an effort to determine the physiological cascade which is responsible for this effect. The polypyrrole modulates signaling pathways of Akt and ERK kinase through their phosphorylation. These effects are related to the presence of low-molecular-weight compounds present in aqueous polypyrrole extracts, determined by mass spectroscopy. The results show that consequences related to the modulation of stem cell differentiation must also be taken into account when polypyrrole is considered as a biomaterial.

## 1. Introduction

The conjugated structure of conducting polymers (CPs) is, among other factors, mainly responsible for their unique properties, such as high electron affinity, low energy optical transmission, and low ionization potential [1]. Another advantageous aspect of utilizing CPs, especially in biomedicine, is their intrinsically combined electron and ionic conductivity. As a result, CPs are able to convert electrical signals to ionic ones and vice versa. This is a crucial factor for the efficient exchange of signals and stimuli between living objects (e.g., cells or tissues) and conducting materials [2]. Therefore, CPs are excellent materials for tissue engineering with respect to stimulating nerve cells and tissues [3], as electrical stimulation increases neurite outgrowth in vitro and promotes nerve regeneration in vivo [4]. Electric stimuli also affect the differentiation of embryonic stem cells (ESCs). In contrast to cells differentiated by growth factors, ESCs stimulated by electric current are able to differentiate into more specific types [5].

Polypyrrole (PPy) is one of the most studied CPs in the context of tissue engineering applications. It can be synthesized by chemical or electrochemical polymerization [6,7]. The electrochemical method is relatively expensive, and, moreover, a conducting substrate is required for the polymerization. The chemical synthesis of PPy can be accomplished using oxidative polymerization, admicellar polymerization, and layer-by-layer deposition [8], giving rise to PPy in the conducting form of a salt, which can be converted by alkali treatment to a non-conducting base [9,10]. PPy polycation is prepared in the form of thin films or powders, and negatively charged counter-ions can be incorporated into its structure during polymerization. By changing the counter-ions, also called “dopants”, the physical properties of the polymer can be controlled [11].

In tissue engineering, the application of electrical stimuli through PPy not only increases neurite outgrowth but also regulates the shape and function of endothelial cells, and enhances the proliferation of smooth muscle cells. PPy also exhibits interesting potential with respect to its application in controlled drug release. If a suitable dopant is incorporated in the polymer, it can be released into the surrounding environment by electrical stimulation [12]. For example, Richardson et al. [13] prepared cochlear implant electrodes coated with a combination of PPy, *p*-toluene sulfonate, and nerve growth factor neurotrophin-3 (NT3). The release of NT3 was controlled by electrical stimuli and neurite outgrowth increased. However, PPy is not only suitable for the cell stimulation by electrical signals, but also exhibits antimicrobial activity. Such antimicrobial activity was, for example, confirmed in a study by Milakin et al. [14], who demonstrated the antimicrobial effect of cryogel scaffolds combining PPy and gelatin.

**Figure 1:**
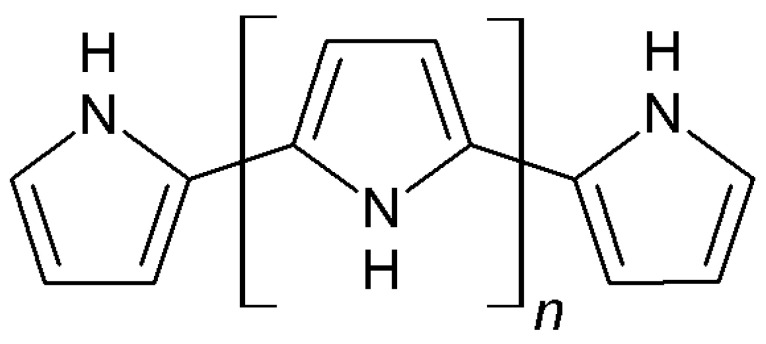
The formula of pyrrole oligomer. *n* = 0, 1, 2, ...

The abovementioned studies clearly demonstrate the unique properties of PPy itself; however, PPy is also interesting due to the properties of its main precursor, heterocyclic pyrrole. The pyrrole molecule is contained in a broad range of therapeutic substances and natural products as one of the main constituents, such as porphyrins. Thus, this heterocyclic compound and its derivatives can serve as the source of a number of pharmacologically active substances. For example, Kang et al. reported on pyrrole-3-carboxamide, which was evaluated as an antidepressant drug [15], and pyrrole derivatives with a sulfonamide group were reported to exhibit antitumor activity [16]. PPy derivatives are also known for their neuroprotectivity. Aiello et al. [17] investigated the natural pyrrole containing product daminin, which showed neuroprotective ability against Parkinson’s and Alzheimer’s diseases. In general, pyrrole derivatives can also act as anti-inflammatory, antituberculous, antiviral, antimalarial, and insecticidal agents [18]. In summary, the above facts suggest that pyrrole and its derivatives affect intracellular signaling pathways. Thanks to the promising application potential of conducting polymers in tissue engineering, the investigation of their biological properties has been at the center of attention for several years. In addition to their basic characteristics, such as cytotoxicity, adhesion, and the ability to enhance cell growth, neurogenesis has remained at the center of efforts towards developing new, smart materials. To the best of our knowledge, no previous study has investigated the effect of PPy, PPy oligomers (Figure 1), low-molecular-weight by-products, or residual impurities present in PPy after its synthesis on the neurogenesis of stem cells. The biological activity of pyrrole and a large number of its derivatives has led the authors to question the role of these low-molecular-weight substances in the control of cellular fate. Therefore, the aim of this paper was to determine the impact of aqueous PPy extracts containing the pyrrole-based substances on the neurogenesis of ESCs.

## 2. Materials and Methods

### 2.1. Preparation of PPy Powders

Polypyrrole salt was synthetized by oxidizing 0.2 M pyrrole (Sigma-Aldrich, China) with 0.5 M iron-(III) chloride hexahydrate (Sigma-Aldrich, Germany) in an aqueous environment. The oxidant-to-pyrrole mole ratio was 2.5. The mixture was left to polymerize at room temperature for 12 h. The precipitated black PPy was collected on a filter, rinsed with 0.2 M hydrochloric acid followed by acetone, and dried at room temperature over silica gel [19]. Preliminary tests conducted in this laboratory demonstrated that the effect of PPy base (formed via the deprotonation of PPy salt with ammonia [10]) on neurogenesis was insignificant; therefore, the present study applies only to PPy hydrochloride (hereinafter referred to as PPy) and the preparation of PPy base is not reported.

### 2.2. Preparation of Extracts

Samples of PPy were extracted according to ISO 10993-12 with the following modification: 0.05 g PPy per 1 mL of cultivation medium was used instead of the ISO-defined ratio of 0.2 g polymer per 1 mL. The extraction was conducted in chemically inert closed containers using aseptic techniques at 37 ± 1 °C under stirring for 24 h. Subsequently, the extract was separated from the polymer powder by centrifugation at 1000 g for 15 min followed by the second centrifugation of supernatant under the same conditions. The parent extracts (100%) were used for the testing of cytotoxicity according to the ISO 10 993-5 protocol. The parent 100% extracts were diluted in complete medium to obtain a series of dilutions. All extracts were used within 24 h. Prior to in vitro testing, the extracts the extracts were sterilized by sterile filtration through a 0.22 μm Millex GV filter (Merck, Darmstadt, Germany). All tests were performed in four separate sets.

### 2.3. Culture of Embryonic Stem Cells

The embryonic stem cell ES R1 line [20] was propagated in an undifferentiated state by culturing on gelatinized tissue culture dishes in complete media. The gelatinization was performed using 0.1% porcine gelatin in water. Complete medium with the following composition was used for the cultivation: Dulbecco’s Modified Eagle’s Medium (DMEM), 15% fetal calf serum, 100 U mL^−1^ penicillin, 0.1 mg mL^−1^ streptomycin, 100 mM non-essential amino acids solution (all from Thermo Fisher, Waltham, MA, USA), 0.05 mM 2-mercaptoethanol (Sigma, St. Louis, MO, USA) and 1000 U mL^−1^ of leukemia inhibitory factor (LIF) (Gibco, MA, USA) [21,22].

### 2.4. Cytotoxicity

The cytotoxicity testing of extracts of PPy was performed according to the ISO 10 993-5 protocol. Samples were extracted according to ISO 10993-12 in the ratio of 0.2 g per 1 mL of DMEM media. Extraction was performed in chemically inert closed containers using aseptic techniques at 37 ± 1 °C under stirring for 24 h. The parent extracts (100%) were then diluted in a complete medium to obtain a series of dilutions with concentrations of 25, 10, 5 and 1%. All extracts were used within 24 h. For proliferation testing, cells were seeded at a density of 5000 cells per cm^2^ 24 h before treatment. As a reference giving 100% cell proliferation, cells cultivated in the complete medium without extract added were used. In addition, the proliferation of cells differentiating in the presence of extracts but without the presence of LIF in the cultivation medium was also tested. Cells were treated with extracts for 48 h and, to assess cytotoxic effects, the mass of viable cells was determined as the level of ATP using Cellular ATP Kit HTS (Biothema, Handen, Sweden). Samples were prepared and analyzed according to Konopka et al. [23]. Before lyses, the morphology of the cells was observed and documented using an inverted Olympus phase contrast microscope (Olympus IX51, Tokyo, Japan) supplemented with a digital camera (Olympus E-450, Tokyo, Japan). All tests were performed in four separate sets. Statistical significance was determined by ANOVA with post hoc Tukey’s Multiple Comparison test; * *p* < 0.05.

### 2.5. ESCs Differentiation

The ESC differentiation was induced through the formation of embryoid bodies (EBs) by hanging drop techniques (400 cells per one 35 μL drop) in LIF-free complete medium [24]. Pictures of growing EBs were captured by digital camera using an Olympus binocular microscope (Olympus SZX7, Tokyo, Japan). The diameter of five-day-old EBs was measured and the number of EBs with expanding neuronal cells was also determined. The EBs were treated with 1, 5, and 25% extract of PPy for 5 days. Consequently, the number of expanding EBs in adherent culture with visible neurites was determined on days 7 and 11 after the seeding of 5-day-old EBs (12 and 16 days of ESC differentiation in total).

**Figure 2:**
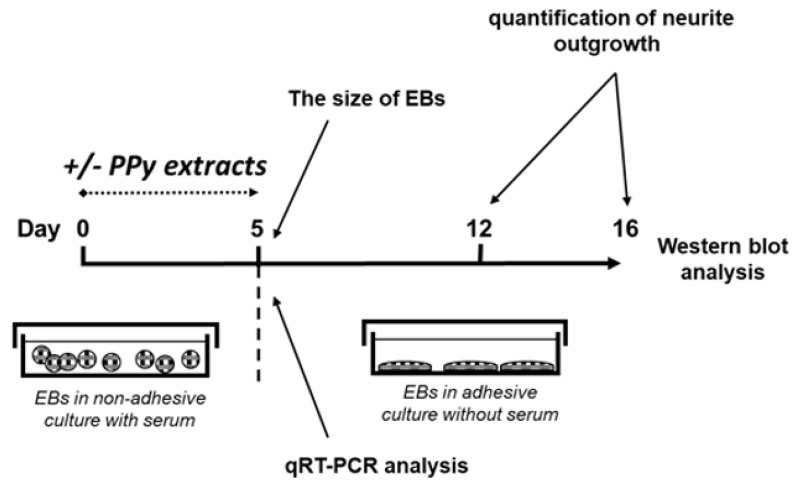
Design of experiment: formation of embryoid bodies in the presence or absence of PPy extracts.

In the next step, the 5-day-old EBs were transferred to a gelatinized 24-well plate (one EB per well) and cultivated in serum-free DMEM-F12 media (1:1) containing 100 U mL^−1^ penicillin, 0.1 mg mL^−1^ streptomycin and insulin–transferrin–selen (ITS) supplement (all from Thermo Fisher, Waltham, MA, USA) for the next 11 days. The medium was replaced with fresh medium every 2 days of cell culture. Differentiating cells were observed using an inverted Olympus phase contrast microscope (Olympus IX51, Tokyo, Japan) and documented with a digital camera (Olympus E-450, Tokyo, Japan) [21]. All tests were performed in four separate sets. Data are reported as the mean and standard deviations from a minimum of four independent experiments. ANOVA with post hoc Tukey’s Multiple Comparison test was applied to determine any statistical differences between the samples. *p* values of ≤ 0.05 were considered statistically significant. Design of experiment leading to formation of EBs in the presence or absence of PPy extracts is given in Figure 2.

### 2.6. Expressions of Neural Markers by Quantitative RT-PCR

Total RNA was extracted by Qiagen RNeasy Mini Kit (Qiagen, Hilden, Germany). Complementary DNA was synthesized according to the manufacturer’ instructions for RevertAid Reverse Transcriptase reverse transcriptase kit (Thermo Fisher, Waltham, MA, USA). Real-time PCR of neural cell phenotype markers was performed in a Roche 480 Light-Cycler using Light Cycler^®^480 DNA SYBR Green I Master (Roche, Basel, Switzerland). Primers and annealing conditions are listed in Table 1. GAPDH was used as a reference gene [24,25,26]. Data are presented as the differences between reference and samples treated with PPy extracts after normalization to the reference gene by the 2^−(Cq(target)−Cq(reference))^ method [27,28]. All tests were performed in four separate sets. Data are reported as the mean and standard deviations from a minimum of four independent experiments. Statistical significance was determined by ANOVA with post hoc Tukey’s Multiple Comparison test; * *p* < 0.05. (*) and (#) mark statistically significant differences compared to ESCs and to EBs without PPy treatment, respectively.

### 2.7. Western Blot

Western blot assay of the expressions of the neuron-specific proteins N-cadherin, N-CAM, β III tubulin, and doublecortin [29,30,31,32,33] in non-differentiating ESCs and in differentiating ESCs with and without PPy treatment (reference (REF)) on Day 16 of differentiation was performed. The level of β-actin was used as a control of protein loading [34]. Cells were directly lysed using 100 mM Tris/HCl (pH 6.8), 20% glycerol, 1% sodium dodecyl sulfate, 0.01% bromophenol blue, and 1% 2-mercaptoethanol. Western blotting was performed according to the manufacturer’s instructions with minor modifications. Concretely, the SDS-PAGE was run at 110 V and the transfer onto polyvinylidine fluoride membrane was conducted for 1 h at 100 V (Mini-Protean III; Bio-Rad, Hercules, CA, USA). Membranes were blocked in 5% non-fat dry milk solution in TBS-Tween 20 buffer (TBST) for 30 min and subsequently incubated overnight at 4 °C with the following primary antibodies: N-CAM (Sigma, St. Louis, MO, USA), N-cadherin (BD Biosciences, Franklin Lakes, NJ, USA, 610920), doublecortin (Abcam, Cambridge, United Kingdom, 18723), β-III-tubulin (Sigma, St. Louis, MO, USA, T5076), p-Akt (phospho-S473; Cell Signaling, 4060), Akt (Cell Signaling Technology, Beverly, MA, USA, 4691), p-ERK1/2 (phospho-T202/Y204; Cell Signaling Technology, Beverly, MA, USA, 8544), ERK1/2 (Cell Signaling Technology, Beverly, MA, USA, 4695), p-GSK3 (phospho-S9; Cell Signaling Technology, Beverly, MA, USA, 9336), and β-actin (Santa Cruz Biotechnology, Dallas, TX, USA, SC47778). The membranes were washed in TBST and incubated with horse radish peroxidase-conjugated secondary antibodies (Sigma, St. Louis, MO, USA). Immunoreactive bands were detected using ECL detection reagent kit (Sigma, St. Louis, MO, USA) and the FusionSL chemiluminescence documentation system (Vilber-Lourmat, Collégien, France). Results were quantified by the densitometric analysis of Western blot bands using the Fiji distribution of ImageJ. All tests were performed in four separate sets. Data are presented as mean and standard deviation from a minimum of four independent experiments. Statistical significance was determined by ANOVA with post hoc Tukey’s Multiple Comparison test; * *p* < 0.05. (*) and (#) mark statistically significant differences compared to ESCs and to EBs without PPy treatment, respectively.

### 2.8. Characterization of Extracts

PPy extracts were analyzed with a 1260 Series liquid chromatography system coupled to a 6520 Accurate-Mass Q-TOF mass spectrometer (both Agilent Technologies, Santa Clara, CA, USA). A dual-spray electrospray ionization source was employed. A sample volume of 5 μL was infused into a continuous flow of mobile phase with no column installed. Samples were eluted with a flow rate of 0.3 mL min^−1^ using 1% (v/v) aqueous formic acid at 30 °C.

Mass spectroscopy analyses were conducted on PPy extracts prepared using the procedure described above, with deionized water as the extraction liquid. The positive ion mode mass spectrometry conditions were as follows: gas temperature, 300 °C; fragmentor voltage, 75 V; capillary voltage, 3 kV; nozzle voltage, 2 kV; scan range m/z, 50 to 1700; 1 scan^−1^. The internal mass reference ions m/z 922.009798 and 121.050873 were used to keep mass axis calibration stable during the analysis.

### 2.9. Statistical Analysis

Statistical significance was determined by ANOVA with post hoc Tukey’s Multiple Comparison test. More detailed information and values of significance are provided in individual chapters in the methodology.

## 3. Results and Discussion

The effect of the electrical conductivity of the material is particularly interesting with respect to excitable tissues and cells, such as cardiac or nerve cells, represented here by neuronal lines derived from ESCs. A prerequisite for using any material in contact with cells and tissues is the absence of its cytotoxicity, which concerns also materials based on PPy. In studies by Humpolíček et al. [19] and Capákova et al. [35], the cytotoxic properties of two PPy forms were tested, namely protonated PPy salt and deprotonated PPy base, which were prepared by various techniques.

As the effect of PPy base on neurogenesis proved to be insignificant, the current study reports only on the investigation of neurogenesis induced by extracts of PPy salt (hereinafter referred to as PPy). As described in the work by Humpolíček et al. [19], PPy extract loses its cytotoxic effect at 5% concentration. In the case of the embryotoxicity of PPy, erythroid cluster formation and beating foci completely disappeared in the presence of 25% extract.

**Figure 3:**
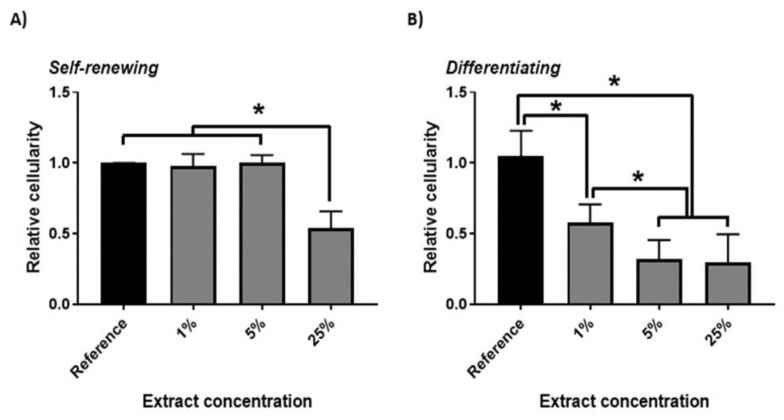
Cytotoxic effect of polypyrrole (PPy) extract on embryonic stem cells (ESCs) after treatment with 1, 5, and 25% extract in self-renewing media (**A**), and in media enabling their spontaneous differentiation (**B**) after 48 h in comparison with reference. Data are reported as the mean and standard deviations from a minimum of four independent experiments. ANOVA with post hoc Tukey’s Multiple Comparison test was applied to determine any statistical differences between the samples. *p* values of ≤ 0.05 were considered statistically significant and are labeled with (*).

The cytotoxic effect of PPy extracts (1, 5 and 25%) on ESCs was also determined in the present study. Cytotoxicity was tested not only on undifferentiated ESCs in the presence of leukemia inhibitory factor (LIF) but also on cells that differentiated spontaneously due to LIF depletion. The results are presented in Figure 3, demonstrating a correlation between relative cellularity, PPy extract concentration and cell phenotype. For undifferentiated cells cultured in the presence of LIF, a cytotoxic effect was observed at the highest tested concentration of PPy extract (25%). The results are comparable with those reported by Humpolíček et al. [19]. The ability of ESCs cultured in the absence of LIF to undergo spontaneous differentiation has already been reported by Smith et al. [36]. In the case of cells that underwent differentiation (Figure 3B), the relative cellularity was reduced by almost 50% already in the presence of the lowest concentration of PPy extract (1%), in comparison with the reference. Therefore, cells cultured under normal conditions, in which they retain their ability to self-renew, and in an undifferentiated state exhibit higher resistance to environmental changes.

**Figure 4:**
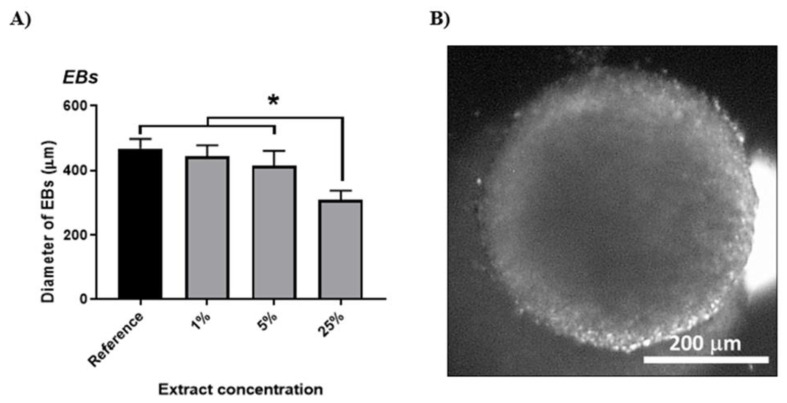
(**A**) Cytotoxic effect of PPy extract on embryoid bodies (EBs). The size of EBs determined on Day 5 of culture. (**B**) Embryonic body. Data are reported as the mean and standard deviations from a minimum of four independent experiments. Statistical significance was determined by ANOVA with post hoc Tukey’s Multiple Comparison test; * *p* < 0.05.

The work described here, which extends the abovementioned study by Humpolíček et al. [19], concentrates on testing the toxicity impact on EBs. Cells were exposed to extracts during the induction and maintenance of EBs for a period of 5 days. In principle, EBs mimic the development of embryos and the cells differentiate into all three embryonic germ layers [37]. The cytotoxic effect of PPy extract on the size of growing EBs is shown in Figure 4A. Significant reductions in EBs sizes were found only in the case of cultivation with 25% extract. Here, the cells differentiated into many types and formed abundant intercellular interactions (Figure 4B). The influence of lower tested concentrations of PPy extract (1 and 5%) did not negatively influence EB dimensions, demonstrating thus no harmful effect on the part of the studied substance.

Due to its conductivity, PPy can serve as smart material influencing cell differentiation and promoting the proliferation of various types of nerve cells. However, additional external factors, such as the presence of signaling molecules or an electric current are needed for successful cell differentiation. Therefore, the use of PPy formulated as a scaffold, which can combine all these factors, enhances the ability of cells to differentiate. A recent study by Granato et al. [38] investigated the differentiation of the murine Neuro2a cell line on poly (butylene adipate-*co*-terephthalate) (PBAT) fibers blended with PPy. The cells were cultured on the material for three days in the presence of media promoting neurodifferentiation. On a substrate containing 2% PPy, the occurrence of neurites was higher in comparison with neat PBAT. The presence of PPy in the blend also increased its hydrophilicity, resulting in improved cell adhesion. In another work, Stewart et al. [39] investigated the effect of PPy on the differentiation of human neural stem cells (hNSCs) in the presence of an electric field. The conducting PPy substrate promoted differentiation into neurons under the influence of electric current, in contrast to the reference (a glass-based substrate with protein laminin). Biocompatibility with Schwann cells is also very important for the use of materials in neural tissue engineering. This was demonstrated in a study conducted by Wang et al. [40], who reported on the absence of cytotoxic effects even in the case of 50% PPy extract. Moreover, Schwann cells were able to proliferate on the PPy membrane. Comparable results were published by Sun et al. [41]. Here, a PPy coated composite of poly (L-lactic acid-*co*-ε-caprolactone) with silk fibroin (PLCL/SF) was used as the test material. In comparison with the reference (non-coated PLCL/SF), Schwann cells manifested better proliferation on PPy-coated PLCL/SF. The authors also used this conducting polymer to induce the differentiation of neuroblastic cells in the absence of nerve growth factor in the culture medium by applying only an electric field. However, they observed that the conducting polymer was not able to induce differentiation without stimulation by the electrical current. On the basis of the findings reported in the above publications, it can be stated that all the mentioned factors playing a role in neurogenesis are mutually supportive.

**Figure 5:**
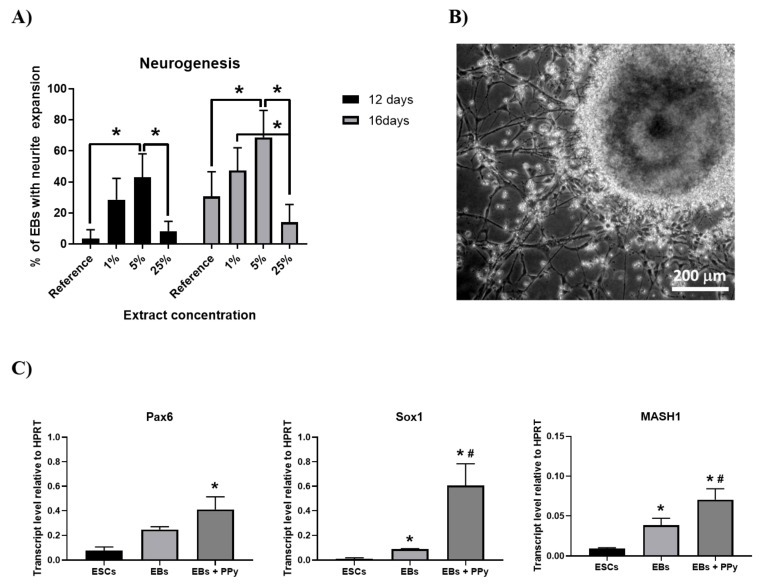
Influence of PPy extracts on neurogenesis. (**A**) Formation of EBs with neurite expansion after treatment with PPy extracts. (**B**) Microscopic visualization of expanded EB with neurites. (**C**) the expression of early neurogenic transcripts (Pax6, Sox1, and MASH1) in non-differentiating ESCs and in 5-day-old EBs growing in the absence (EBs) or in the presence of 5% PPy extract (EBs + PPy). Data are reported as the mean and standard deviations from a minimum of four independent experiments. Statistical significance was determined by ANOVA with post hoc Tukey’s Multiple Comparison test; * *p* < 0.05. (*) and (#) mark statistically significant differences compared to ESCs and to EBs without PPy treatment, respectively.

However, to date, no studies have been published on the effect of PPy extracts on the neurogenesis of ESCs. Here, EBs were formed by the hanging drop method in the presence of 1, 5 and 25% PPy extracts for 5 days. Then, EBs were cultivated on adherent plastic in the absence of serum in the culture medium. Neurogenesis was evaluated on days 7 and 11 of culture (12 and 16 days of overall differentiation, respectively) as the percentage of bodies with visible neurites (Figure 5B). Compared to reference cells, extract-treated EBs developed earlier and more frequent neuronal processes. The 5% PPy extract exerted the greatest effect on the formation of neurites (Figure 5A). In the light of these results, the expressions of early neurogenic transcripts were evaluated in 5-day-old EBs growing in the presence of 5% PPy extract. For comparison, expression was also assessed in undifferentiated ESCs and EBs formed without extract. PAX6 [42], SOX1 [43] and MASH1 [44] are factors important for neurogenic processes and are expressed primarily at the beginning of neuron development. As expected, the expression levels of these transcripts in ESCs were very low. EBs cultured in the absence of extract increased the expression of all genes due to spontaneous differentiation. However, the highest level of expression was observed in EBs in the presence of 5% PPy extract (Figure 5C).

Neurogenesis was also determined through the protein level by means of Western blot assay. The neuron-specific proteins were carefully chosen on the basis of their properties and action during neurogenesis. N-cadherin was one of the neuroproteins tested in the present study. N-cadherin has an important function for cell adhesion and also for cell interactions under the development of the central nervous system (CNS) [29]. N-CAM (also known as CD56), the neural cell adhesion molecule, was chosen as another marker of neurogenesis. This immunoglobulin occurs on the cell surface in the central and peripheral nervous systems. Along with N-cadherin, it is involved both in cell adhesion and in synaptogenesis [30]. A protein that is also involved in neurogenesis is β-III-tubulin (or TUJ1). It is predominantly expressed in the cytoskeleton of neuroblasts [31]. Another protein detectable under the development of embryonic neural systems is doublecortin (doublin), which belongs to the family of microtubule-associated proteins (MAPs) and is mainly involved in neuronal migration [32,33]. It is commonly used in experiments as one of the markers of neurogenesis.

**Figure 6:**
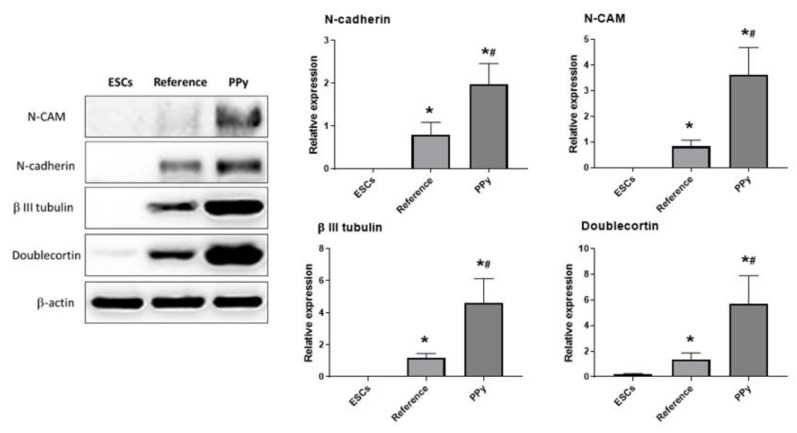
Western blot assay of the expression of neuron-specific proteins N-cadherin, N-CAM, β-III-tubulin, and doublecortin in non-differentiating ESCs and in differentiating ESCs with PPy treatment (PPy) and without PPy treatment (Reference) on day 16 of differentiation. The level of β-actin was used as a control of protein loading. Data are reported as the mean and standard deviations from a minimum of four independent experiments. Statistical significance was determined by ANOVA with post hoc Tukey’s Multiple Comparison test; * *p* < 0.05. (*) and (#) mark statistically significant differences compared to ESCs and to Reference, respectively.

In the present study, the expressions of the abovementioned neurospecific proteins (N-cadherin, N-CAM, β-III-tubulin, and doublecortin) were studied, with beta-actin used as a reference protein. Expression was determined after 16 days of cultivation both in undifferentiated ESCs and in differentiated cells in the absence and presence of 5% PPy extract. The results of the assay, summarized in Figure 6, show a significant increase in the expressions of all the tested neuroproteins in PPy-treated cells, these being higher by 50% or more than in the samples with PPy extract absent. The presented observations, therefore, indicate that PPy extracts contain compounds that positively affect cell neurogenesis.

Akt (or PBK) is a serine/threonine kinase involved in a variety of processes such as the regulation of cell growth, proliferation, migration, protein synthesis, and differentiation. The disruption of Akt kinase-mediated signal transduction leads to neurodegenerative diseases, such as Alzheimer’s, Parkinson’s, and Huntington’s disease. Akt occurs in the organism in three forms (Akt1, Akt2 and Akt3) that share a highly conserved structure. All isoforms are abundantly expressed in the CNS and their activation is affected by various growth factors, insulin, or cell stress. Akt activity is determined as the ratio of phosphorylated Akt at Ser473 to total Akt [45,46]. Through the PI3K signaling pathway, Akt is involved in neuronal survival and growth [47,48]. GSK-3 (glycogen synthase kinase-3) is one of the most important physiological substrates for Akt [49,50]. The activity of this signaling molecule is usually inhibited by Akt phosphorylation, following a response to stimulation by a certain factor [51,52].

**Figure 7:**
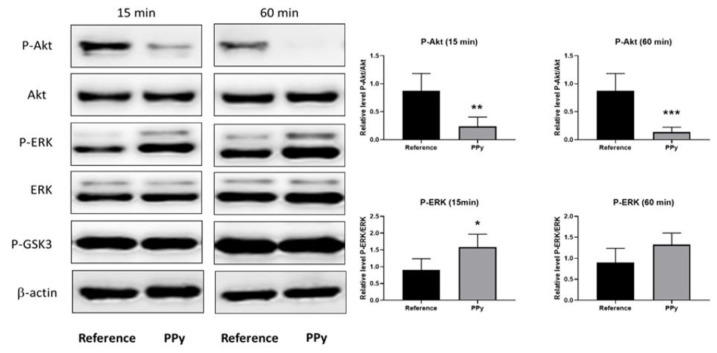
PPy extract inhibits Akt kinase phosphorylation. ESCs were either exposed or not exposed (reference) to 10% PPy extract (PPy) for 15 and 60 min. The levels of active, phosphorylated forms of Akt, ERK and GSK3 kinases, and the level of β-actin were determined by Western blot techniques. The level of β-actin was used as a control of protein loading. Representative Western blot and quantified data from four independent experiments are shown. Statistical significance was determined by *t*-test; * *p* < 0.05; ** *p* < 0.01; *** *p* < 0.001.

To discover possible molecular mechanisms underlying the influence of PPy extract on neurogenesis, the status of selected signaling proteins was determined (Figure 7). Various pyrrole-containing compounds have the ability to inhibit the activity of GSK3 [53,54,55]. Other kinases, such as Akt (or PKB) and/or ERK can phosphorylate and thus also modify the activity of GSK3 [56,57]. The ability of PPy extract to inhibit the phosphorylation of the mentioned kinases was also determined within this study. Concretely, the ESCs were seeded 24 h before treatment with 10% PPy extract and subsequently exposed to extract for 15 and 60 min. Cells were lysed and analyzed by the Western blot technique. PPy extract significantly attenuated the level of phosphorylated Akt kinase at both tested time points. In contrast to Akt, the level of phosporylated ERK increased; however, a significant increase was observed only in the sample treated for 15 min. No changes were observed in the phosphorylation of GSK3 in cells treated by PPy extract (Figure 7). As already mentioned above, it was reported that various pyrrole-containing compounds inhibited the activity of GSK3 [53,54,55]. GSK3 plays an important role in a myriad of cellular processes including the regulation of neurogenesis and neuronal function in the developing and adult brain [57]. The phosphorylation status of GSK3 Ser9 was unchanged in cells exposed to PPy extract. Accordingly, we believe that the activity of GSK3 was not modified by extract of PPy. Unexpectedly, here, downregulation of the phosphorylation of GSK3 upstream kinase Akt (Ser473) was observed, which reflects the inhibition of Akt activity. In contrast, the phosphorylation of ERK (another important upstream kinase of GSK3) was slightly downregulated. This reciprocal phospho status of Akt and ERK kinases corresponds to mutual competition between their signaling pathways [58]. The stimulation of ESC neurogenesis by the inhibition of Akt kinase activity was described previously [59]. Moreover, many growth factors activate Akt signaling in many cell types. On the other hand, growth factor depletion, which also leads to the inhibition of Akt activity, provides the initiation of neurogenesis in ESCs [60,61]. On the basis of these facts, we assume that the promotion of neurogenesis in ESCs by PPy extract is connected with the PPy extract-mediated modulation of Akt signaling pathway activity.

Work undertaken to determine the reasons for the cytotoxicity of polyaniline was previously published by Stejskal et al. [62] and Kašpárková et al. [63]. On the basis of their findings, it can be assumed that such cytotoxic effects are mainly connected with the presence of low-molecular-weight impurities. In order to look into the composition of PPy extracts and to identify any low-molecular-weight products and oligomers that might induce neurogenesis, electrospray mass spectrometry (MS) analyses of the samples were conducted.

**Figure 8:**
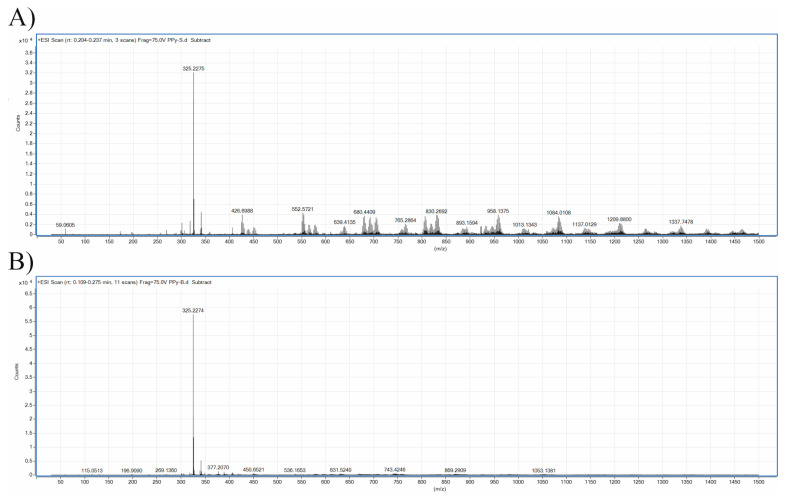
Positive-ion ESI-MS spectrum of polypyrrole extract; (**A**) polypyrrole salt, (**B**) polypyrrole base.

Mass spectrometry, with the exception of pyrolysis MS, has not frequently been used as an analytical tool for the characterization of PPy. This is mainly due to the extremely poor solubility of PPy in common solvents suitable for MS. However, the studied aqueous PPy extracts, which are assumed to contain low-molecular-mass fractions/impurities of the polymer, can be accessible for analysis. A representative mass spectrum of the studied PPy extract is shown in Figure 8A, and, from this, it is obvious that within the studied m/z region, the sample comprises one main dominating molecular ion (M + H) + m/z = 325.2. Higher masses are also present at significantly lower intensities, with a pattern typical for oligomers. The interpretation of the recorded MS spectra is not, however, straightforward and, surprisingly, very limited information on the occurrence or structure of PPy oligomers and impurities exists in the literature [64,65]. Nevertheless, the mass of the main molecular ion with m/z = 325.2 can be assigned to PPy pentamer. Higher masses can then correspond to oligomers and/or their adducts, and may comprise oxygen and oxygen containing by-products. Taking into consideration the presence of Cl^−^dopant anions originating from FeCl_3_ oxidant, the presence of adducts containing chlorine can also be expected. Interestingly, the higher masses present in the spectrum are separated by a mass of about Δm/z ~125 Da. A similar observation was reported by Appel et al., who studied oligomers soluble in a PPy reaction mixture using UV–Vis and GPC techniques [64]. In their work, PPy was synthetized in two ways: (1) from *p*-toluene sulfonic acid and pyrrole in acetonitrile via oxidation by oxygen in the surrounding air, and (2) electropolymerization from the same system. The authors concluded that, in both cases, there are some preferred oligomers formed with *n* = 3, 4, 7, 12 and 19, and that oligomers up to *n* = 30 were present. This is an indication of the formation of larger oligomers by combinations of smaller ones. In addition, Fermin and Scharifker [65] studied oligomers under elctropolymeration with aqueous KNO_3_ or KCl as electrolytes and observed soluble oligomers with up to nine pyrrole units in the electrolyte solutions, together with polypyrrole gradually depositing onto the electrode surface. Though neither of the reported procedures can be compared with oxidation using FeCl_3_, it is quite obvious that the presence of different oligomer types will depend on the polymerization procedure and oxidation agent, as well as the reaction system. The interpretation of the spectra is also complicated because of the known shortcomings of ESI-MS—namely, the fact that analytes with higher molecular masses may acquire several electrostatic charges during the ESI process. A series of multiply charged species are consequently produced, including adducts with impurities, water, and components of mobile phase [66]. Unambiguous deconvolution of the spectra recorded for PPy extract in our work is, therefore, difficult and is beyond the scope of the current study.

Combining the results from MS analysis and cytotoxicity testing, it can be concluded that neither of the detected substances exhibit severe cytotoxicity to EBs, as shown by their insignificant size reduction after treatment with 5% PPy extract. Neurogenesis, however, is induced by species with m/z higher than 325.5, as this pentamer is still present in the deprotonated PPy base (Figure 8B), which, in the present study, have not induced neurogenesis. The oligomers with higher masses are absent or detected in substantially lower amounts. The absence of higher oligomers in the PPy base is due to the deprotonation process transforming PPy from salt to base form, which serves as a purification procedure. There are a number of substances which can be present as by-products of PPy synthesis and can regulate stem cell behavior. In this respect, the work of Park et al. [67] can be mentioned. In this study, the influence of extract of dried Euphoria longan, which has been used in ancient Chinese medicine for the treatment of forgetfulness, was tested. The extract was tested in vivo in mice and it was shown that it regulates neurogenesis and promotes memory. A pyrrole-lactone-based natural alkaloid named longanlactone was later isolated from Euphoria seeds [68]. This pyrrole-lactone is responsible for the promotion of neurogenesis, as confirmed in a recent study by Reddy et al. [69], in which various synthetic derivatives of longanlactone have also been tested.

## 4. Conclusions

The conjugated structure of conducting polymers (CPs) is, among other factors, mainly responsible for their unique properties.

Polypyrrole, an example of a conducting polymer, is one of the most attractive materials within this group for applications in tissue engineering. It finds application not only because of its good level of biocompatibility, but also due to its intrinsic combination of electronic and ionic conductivity. These characteristics are mainly advantageous when preparing biointerfaces, and controlling and regulating cell fate. PPy itself, as a chemical substance, exhibits no known effects on the differentiation of stem cells. However, the question remains whether and to what extent any of the low-molecular-weight products and residual impurities present in PPy affect cell physiology. In the present study, the cytotoxicity of aqueous PPy extracts and their effects on the differentiation of embryonic bodies formed from mouse embryonic stem cells (ES R1 line) were investigated. Cytotoxic effects on undifferentiated cells cultured in the presence of leukemia inhibitory factor were first observed at the highest tested concentration of PPy extract (i.e., 25%). On the other hand, in cells that underwent differentiation, relative cellularity decreased by ~50% relative to the reference already after treatment with the lowest extract concentration (i.e., 1%).

With regard to neurogenesis, EBs cultured in the presence of PPy extracts (especially those with 5% concentration) formed nerve processes and amplified neurite expansion more abundantly than non-treated EBs (reference). In the light of these findings, the expressions of genes associated with early neurogenesis (specifically Pax6, Sox1, and MASH1) were measured in EBs formed in the presence of 5% PPy extract and found to be higher than in the reference EBs. Similarly, the expressions of neurospecific proteins (N-cadherin, N-CAM, β-III-tubulin, doublecortin) increased in comparison with the reference. The 10% PPy extract then exerted an effect on the modulation of Akt and ERK kinase signaling pathways. This effect is also associated with neurogenesis. The observed induction of neurogenesis in EBs could be related to the presence of PPy oligomers in the extract, which were determined by mass spectrometry. The cellular behavior is likely influenced by these low-molecular-weight compounds; however, the exact determination of which of these compounds causes this effect is beyond the scope of this study.

## Figures and Tables

**Table 1:** Primer sequences for target and reference genes used in qRT-PCR assays; F = forward primer (5′–3′), R = reverse primer (3′–5′).

Gene	Primer Sequence	T annealing (°C)	Product Size (pb)
*Pax 6*	F TGCCCTTCCATCTTTGCTTG	60	178
	R TCTGCCCGTTCAACATCCTTAG		
*Sox 1*	F CCAGCCTCCAGAGCCCGACT	62	258
	R GGCATCGCCTCGCTGGGTTT		
*Mash 1*	F GGTCTCGTCCTACTCCTCCG	62	137
	R GCTGCCATCCTGCTTCCAAA		
*Gapdh*	F AAGGGCTCATGACCACAGTC	62	252
	R CATACTTGGCAGGTTTCTCCA		

## Data Availability

The data presented in this study are available on request from the corresponding author.

## Abbreviations

CPs	conducting polymers
ESCs	embryonic stem cells
PPy	polypyrrole
NT3	neurotrophin-3
LIF	leukemia inhibitory factor
EBs	embryoid bodies
ITS	Insulin–transferrin–selen
REF	reference
PBAT	butylene adipate-*co*-terephthalate
hNSCs	human neural stem cells
PLCL/SF	composite of poly (L-lactic acid-*co-ɛ*-caprolactone) with silk fibroin
CNS	central nervous system
MAPs	microtubule-associated proteins
GSK-3	glycogen synthase kinase-3
MS	mass spectrometry
